# Computerized Platform in Hindi Language: Exploring Its Use in Aphasia Screening

**DOI:** 10.1109/JTEHM.2026.3733827

**Published:** 2026-09-14

**Authors:** Paromita Mitra, Shipra Kethwalia, Rhythm Makkar, Ruchita Hazrati, Rahul Chhatlani, Geetesh Kumar Singh, Ashima Nehra, Uttama Lahiri

**Affiliations:** Department of Electrical EngineeringIIT Gandhinagar Gandhinagar 382055 India; Clinical Neuropsychology DepartmentNeurosciences CentreAll India Institute of Medical Sciences 28730 New Delhi 110029 India; Shree Aryatej Institute of Physiotherapy Morbi 363642 India; Rastriya Raksha University Dahegam Gujarat 382305 India

**Keywords:** Aphasia, stroke, interbeat interval, response time, western Aphasia battery

## Abstract

Objective: Aphasia is a frequent sequela of stroke, affecting approximately 25%–40% of stroke survivors due to focal damage to language-related brain regions. In resource-constrained settings like India, timely screening is limited by the cost of neuroimaging and the time-intensive nature of conventional pen-and-paper assessments. This study presents a Computerized Hindi Aphasia Screening Platform offering various tasks (CHASP), a computer-based multimodal screening platform designed to objectively differentiate aphasic and non-aphasic post-stroke individuals from healthy controls. Methods: CHASP integrates Acoustic, and Visual and Reading tasks with a sensor-enabled base station for response-time quantification and a physiology-based module for acquiring photoplethysmography-derived interbeat interval (IBI) as a biomarker of cognitive processing. Post-stroke participants were independently assessed using the Western Aphasia Battery (WAB), the gold-standard clinical tool. Performance scores from CHASP and relevant sections of WAB were compared for exploring clinical relevance. Results: The CHASP task modules reliably differentiated post-stroke participants from healthy controls across performance scores, response times, and IBI. The Acoustic tasks could distinguish the aphasic and non-aphasic groups, particularly through task scores and IBI measures. Strong correlations were observed between CHASP performance scores and corresponding WAB scores, supporting the clinical validity of the proposed platform.

Clinical and Translational Impact Statement:—Computerized Hindi Aphasia Screening Platform, a computer-based multimodal platform quantifying performance, response time biomarker variations corresponding to tasks facilitating screening of aphasic post-stroke individuals for timely intervention and clinical translation.

## I. Introduction

APhasia, an acquired language disorder resulting from focal brain damage is characterized by impairments in language production and comprehension [1], [2]. Among many causes of Aphasia, stroke is a prominent cause accounting for nearly 25%–40% of the cases having Aphasia [3]. The situation is grim in India which reports nearly 1.5–2 million new stroke cases annually with an estimated 0.6–0.8 million new Aphasia cases each year [4].

Intervention holds potential in altering recovery trajectories through timely therapies [5], [6], necessitating timely screening of cases of Aphasia. Detection of Aphasia traditionally relies on use of neuroimaging modalities [7] which are often expensive to avail and can be accessed only in specialized urban-based pockets [8], [9]. Another modality, namely International Classification of Functioning (ICF), Disability and Health framework has been used to screen Aphasia based on impairment-level, activity-level, and participation-level domains [10]. Alternately, pen-and-paper-based neuropsychological assessments are widely used. For example, the Western Aphasia Battery (WAB) is considered as one of the gold-standard tools for assessing Aphasia and quantifying its severity through standardized task-based scoring [11]. Again, given that Aphasia is related to language disorder, pen-and-paper based assessment tools in various languages are being used, e.g., the Chinese Rehabilitation Research Center Aphasia Examination for individuals who are Chinese-dialect-speaking, the Aachen Aphasia Test for German-speaking individuals, the Boston Diagnostic Aphasia Examination for individuals who are English-speaking, the Arabic Diagnostic Aphasia Battery, for those who use Lebanese-Arabic dialect [12], the Indian Aphasia Battery for those who are Hindi-speaking [13], etc. Such pen-and-paper-based tools though effective, primarily generate performance scores in different tasks and do not offer other metrics e.g., response time (i.e., time taken to respond to a task). Also, such assessments are time-intensive, often requiring multiple visits for comprehensive assessment, resource-intensive, etc. [14], [15]. These limitations emphasize the need for scalable screening alternatives, so that once screened, the patient can be referred to a clinician for further assessment.

In response to this need, computerized screening platforms have emerged as complementary tools in the hands of clinicians enabling rapid and scalable screening of Aphasia. Thus, research has been focused on developing tablet and computer-based batteries, and telehealth-enabled assessments. For example, there are computerized screening platforms set in specific languages and adapted from the currently-existing pen-and-paper based assessment tools. Specifically, the Russian Aphasia Test (RAT) [16] is a computerized assessment instrument in Russian language with structure and materials being adapted from the Quick Aphasia Battery (QAB) framed in English [17]. Also, the electronic Comprehensive Aphasia Test (eCAT) is a direct digital version of the established Comprehensive Aphasia Test (CAT), both using the medium as English [18]. None of the computerized assessment platforms use Hindi. Again, there are computerized screening platforms that have not been adapted from the currently-existing pen-and-paper based assessment tools, such as the Short and Tailored Evaluation of Language Ability (STELA) [19], Access2Aphasia^TM^ [20], AutoRAT [16], etc. that are framed in Japanese, English and Russian language respectively. Specifically, the STELA is a tablet-based system used for assessing auditory and reading comprehension, naming, repetition, etc. using touchscreen-based interaction [19]. Again, the Access2Aphasia^TM^ is an iPad-based application that supports remote assessment [20]. Also, the AutoRAT uses automated scoring mechanisms to enable quantification of one’s language impairment [16]. Though powerful, all of these existing computerized screening platforms primarily generate only performance scores and do not offer other metrics e.g., response time, variations in biomarkers (such as pulse rate) while interacting with the tasks (and sensed using a computerized platform [21]) which can provide insights into the underlying cognitive processing involved in making a decision before responding to a task [22].

Thus, there exists a critical need to come up with a computerized screening platform that can (a) be augmented to quantify one’s performance in a task in terms of scores, response time and variations in biomarkers and (b) offer stimuli in Hindi given that nearly half of the population in India are native Hindi speakers [23]. Motivated by this need, we have come up with a Computerized Hindi Aphasia Screening Platform (CHASP) adapted from the Indian Aphasia Battery (IAB) [13]. The CHASP was used to offer audio and visual comprehension tasks and capture one’s task performance in terms of performance scores, response time, and variation in biomarker while one responds to the task. The objectives of our study were to (i) design the CHASP platform and (ii) carry out a study to explore the use of the CHASP platform in differentiating (a) post-stroke and healthy groups to start with followed by (b) Aphasic and Non-Aphasic groups. Further, given that the CHASP was designed as a computer-based adaptation of the IAB, for understanding the clinical relevance of CHASP, we chose the relevant sections of the WAB which is considered as one of the gold-standard tools for assessing Aphasia [11]. The rest of the paper is organized as follows: Materials and Methods are presented in Section II, which is subdivided into *System Design* and *Experiment and Methods,* followed by Results and discussions in Section III and finally Conclusion in Section IV.

## II. Material and Method

### A. System Design

In our present work, we have developed a Computerized Hindi Aphasia Screening Platform using Python environment offering audio and visual comprehension tasks adapted from the Indian Aphasia Battery (IAB) [13] (widely practiced by neuropsychologists in India working with speech and language disorders). The CHASP comprised (1) a Computer-based task, (2) a Sensored Base Station and (3) Physiology-based Data Acquisition Module (Fig. 1). The computer-based task module offered various types of tasks. The Sensored Base Station module quantified one’s time taken to initiate a task response. The Physiology-based Data Acquisition module acquired one’s physiological signal during task execution.

**FIGURE 1.**
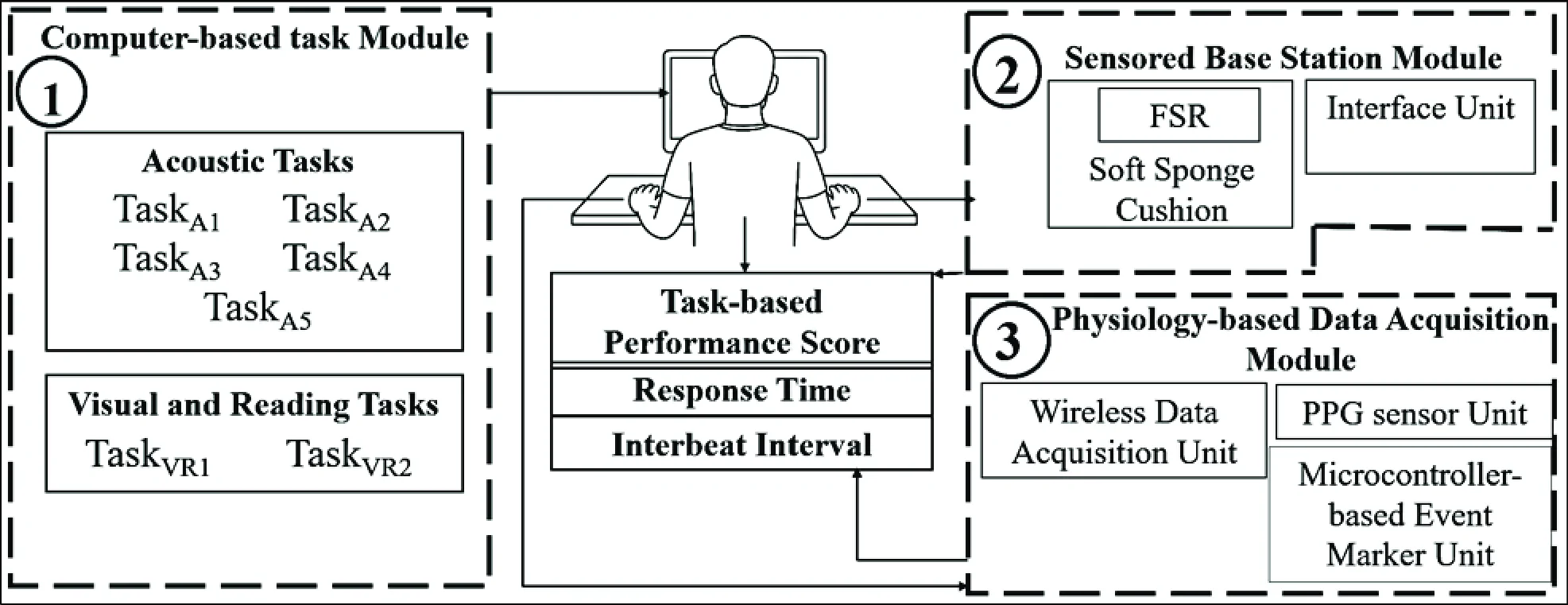
Bird’s eye-view of the computerized hindi aphasia screening platform. Note: ‘Task_A1_’ to ‘Task_A5_’ - sub-tasks of acoustic task; and ‘Task_VR1_’ and ‘Task_VR2_’ - subtasks of visual and reading task.

#### 1) Computer-Based Task Module

This module presented an initial dashboard displaying two types of tasks, namely (a) Acoustic, and (b) Visual and Reading tasks with each comprising various sub-tasks. In each task, a stimulus was presented on the display screen of a Task Computer, along with ‘Replay’ and ‘Home’ buttons for replaying a question (in audio mode) and returning to the main dashboard, respectively.

##### a) Acoustic tasks:

These tasks were aimed towards quantifying one’s auditory comprehension ability, which is adversely affected in those with Aphasia [13]. This comprised of five audio-visual tasks (Fig. 2) e.g., (i) Identifying common words (Task ${}_{\mathrm {A1}}$), (ii) Distinguishing between paired words (Task ${}_{\mathrm {A2}}$), (iii) Understanding the meaning of sentences (Task ${}_{\mathrm {A3}}$), (iv) Following directions (Task ${}_{\mathrm {A4}}$) and (v) Understanding a passage (Task ${}_{\mathrm {A5}}$) using pre-recorded audio file.

**FIGURE 2.**
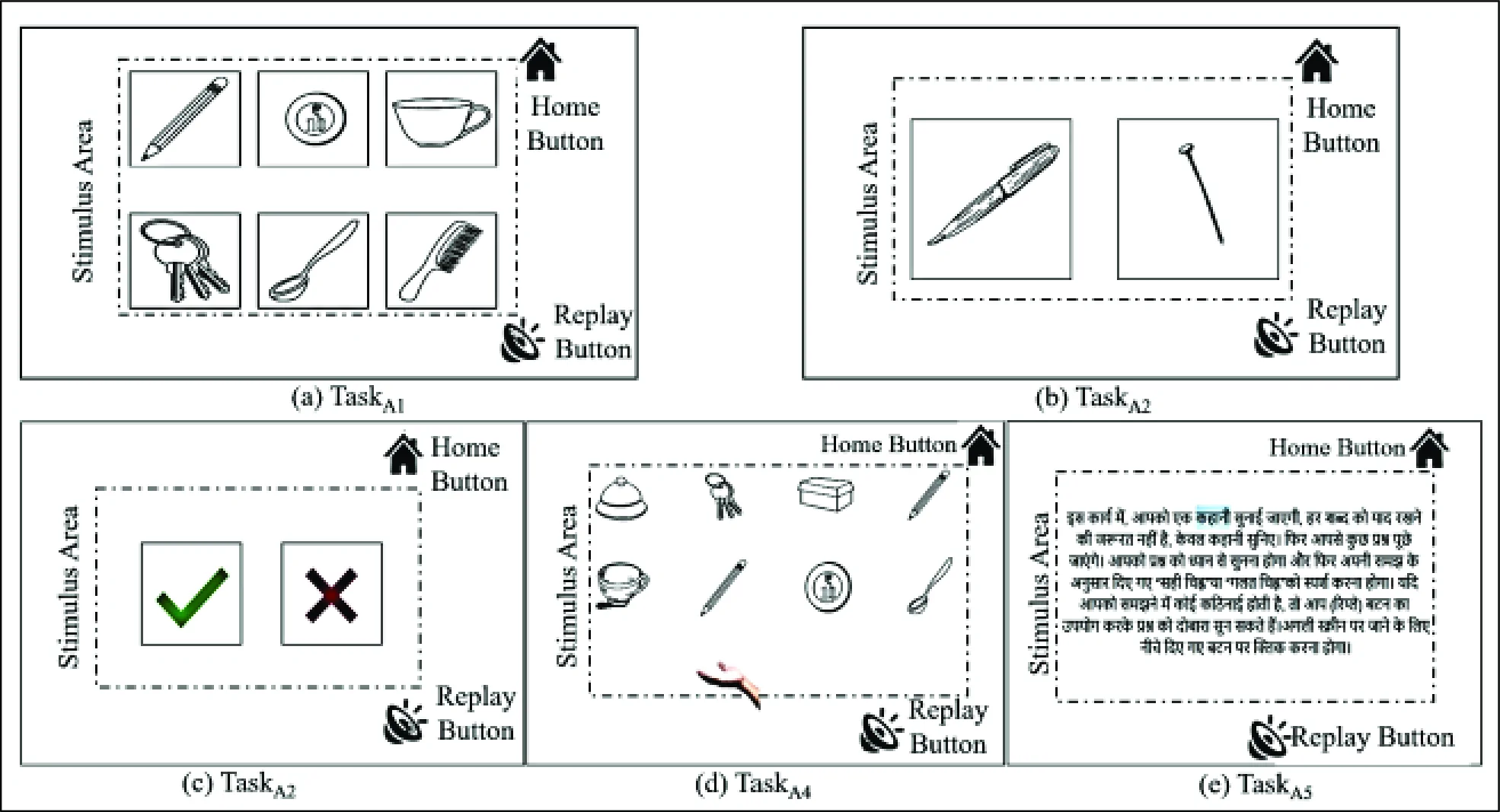
(a) Task_A1_ (Identifying common words, (b) Task_A2_ (Distinguishing paired words), (c) Task_A3_ (Understanding the meaning of sentences), (d) Task_A4_ (Following directions) and (e) Task_A5_ (Understanding a passage).

The Task_A1_ consisted of 3 sub-tasks with each presenting (i) a stimulus having images of 6 objects (line image in black against a white background, similar to another study [21]) chosen in a randomized manner from a database of 18 images (of daily-living objects used in the IAB battery and the images being designed in close consultation with linguists, artists and psychiatrists involved in the administration of the IAB) followed by (ii) a question (in audio mode) asking one to choose an object (out of the 6 objects). Fig. 2(a) shows a typical example of one sub-task in Task_A1_ in which one was asked to choose ‘*cup*’ out of the given 6 images by touching the image on the display screen. Upon one’s response, an audio message was offered to reinforce one for the following task (similar to that used by neuropsychologists working with the target group). Similar steps were followed for all the sub-tasks.

Again, the Task_A2_ consisted of 10 sub-tasks with each presenting (i) a paired similar-sounding stimulus images chosen in a randomized manner from a database of 20 images (ten pairs of daily-living objects used in the IAB battery), followed by (ii) a question (in audio mode) asking one to choose one of the objects. Fig. 2(b) shows an example in which one was asked to choose ‘*Pin*’ among the two displayed images of ‘*Pen*’ and ‘*Pin*’. The Task_A3_ consisted of 10 sub-tasks, with each offering (i) a question (in the audio mode) related to commonly known facts, followed by (ii) displaying images of a tick and cross (for ‘*ye*s’ and ‘*no*’, respectively). The Fig. 2(c) shows the ‘*Tick*’ and ‘*Cross*’ options, while the question, for example was whether the Sun rises in the East. Again, the Task_A4_ consisted of 8 sub-tasks with 3 sub-tasks in difficulty level 1, 3 in level 2 and the rest in level 3 with the level of difficulty being based on the number (i.e., 1, 2, or 3) of objects to be chosen from a stimulus screen presenting (a) 8 different objects of common use (as used in IAB) and an image of a palm (of a hand). The purpose of using the palm image was to simulate a scenario of assessment (used in conventional settings) wherein a clinician asks an individual to hand over specific objects (as named by the clinician) from among 8 different objects presented on a table. The Task_A4_required 2 types of responses, e.g., ‘screen-touch’ or ‘drag-and-drop’ to the palm (image) from each user based on the question being asked. For example, if the question was ‘Ring the bell, close the box and handover the ‘*key*’ (Fig. 2(d); a sub-task in difficulty level 3), then one was required to use ‘screen-touch’ option for the ‘*bell*’ (when the ‘*bell*’ image changed to the image of a ‘*ringing bell*’ and ‘*box*’ (when the ‘*box*’ image changed to the image of an ‘*open box*’) images and ‘*drag-and-drop*’ option for the ‘*key*’. One could use any order in choosing objects. Finally, the Task ${}_{\mathrm {A5~ }}$ offered (i) a stimulus screen with one passage (in Hindi text) presented audio-visually along with highlight-based assistive reading (synchronized with audio input) followed by (ii) questions based on the passage. The response was collected from one’s choice of ‘*Tick*’ or ‘*Cross*’ image (‘*True*’ or ‘*False*’, respectively).

##### b) Visual and reading tasks:

The Visual and Reading task module was aimed to assess the visual comprehension ability. This comprises of 2 visual tasks, e.g., (i) Word-Picture match (Task ${}_{\mathrm {VR1}}$) and (ii) Comprehending passage (Task ${}_{\mathrm {VR2}}$).

The Task_VR1_ consisted of 16 sub-tasks, with each presenting a stimulus having one image (similar to that in Section II-A1) and a pair of written words (with changes in the order of syllables constituting the word conveying different items or objects). Fig. 3(a) depicts a typical example wherein an image of a girl is presented along with two words, ‘
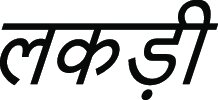
’(meaning Wood) and ‘
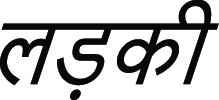
’ (meaning Girl) which sounds as Lakdi and Ladki (in English) to simulate the audio-mode version of ‘
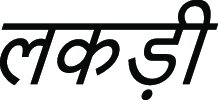
” and ‘
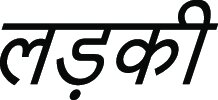
”, respectively. For response, one needed to use ‘*screen-touch*’ to choose the relevant word. The Task_VR2_ presented (i) a stimulus screen with one passage (in Hindi), e.g., in Fig. 3(b), followed by (ii) questions based on the passage. The response was collected from one’s choice of ‘Tick’ or ‘Cross’ images (similar to Task_A5_; Section II-A.1).

**FIGURE 3.**
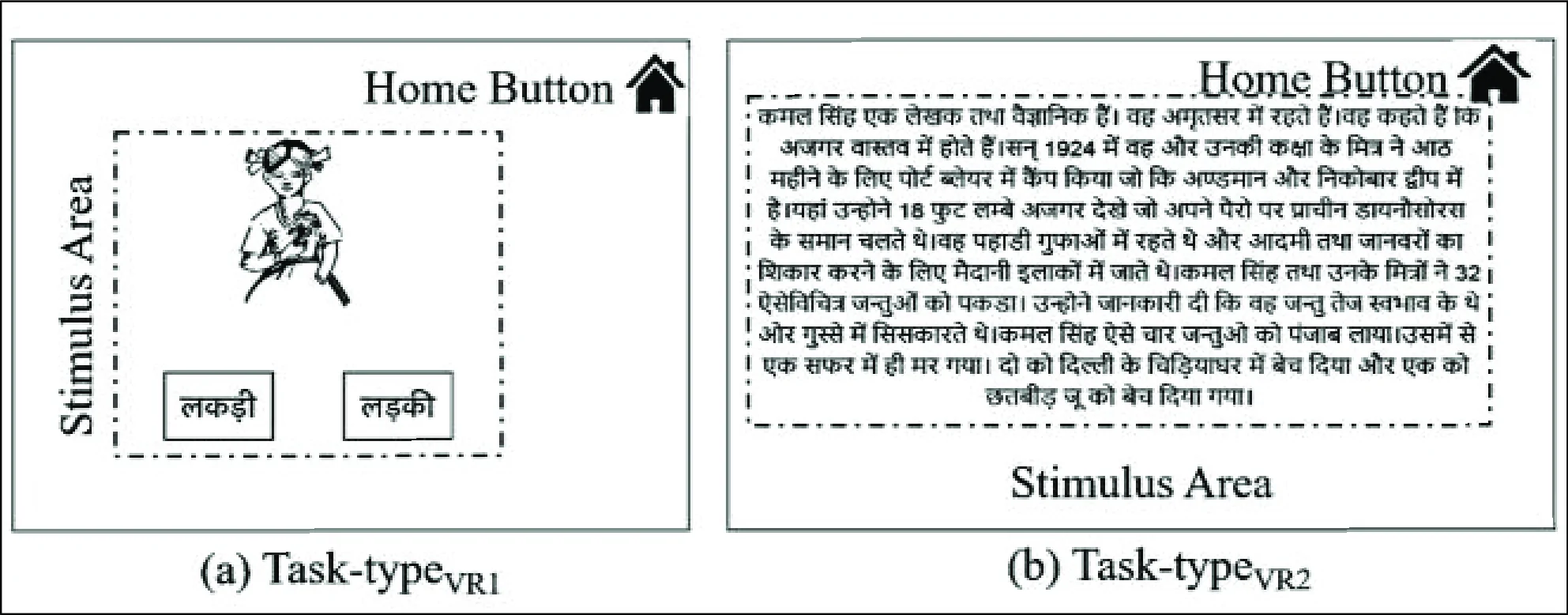
(a) Word-Picture match (Task ${}_{\mathrm {VR1}}$) and (b) Comprehending passage (Task ${}_{\mathrm {VR2}}$).

#### 2) Sensored Base-Station Module

The CHASP comprised a sensor-enabled Base Station that was used to quantify the time one needed to initiate a response in a task (Section II-A). This consisted of (i) a force-sensitive resistor (FSR; 0.1 N- 10 N [24]) that was embedded in a cushion-like pad (on which one can rest hand) firmly attached to a surface (Fig. 1) and (ii) an interface unit that integrated the FSR to the Task Computer storing the time-stamped hand-status information (‘*1*’ for hand on pad and ‘*0*’ for hand not on pad) corresponding to each sub-task. Subsequently, based on the time stamps, the CHASP was used to quantify one’s Response Time (Section III-A.2).

#### 3) Physiology-Based Data Acquisition Module

This module (Fig. 1) of the CHASP was aimed to acquire one’s biomarker, based on the Photoplethysmogram data. This module consisted of (i) Biopac MP150 [25] with wireless data acquisition at a sampling rate of 1000 samples/sec, (ii) a Photoplethysmogram (PPG) sensor along with the transducer (that comes with Biopac) and (iii) a microcontroller-based event marker unit. The data acquired from the PPG sensor was routed by the transducer to the backend database of the CHASP via Biopac MP150 transmitter unit. Simultaneously, based on the events in a task execution, e.g., task presentation, one’s response, etc., the microcontroller-based event marker unit was used to log event markers in the backend of the CHASP.

### B. Experiment and Method

#### 1) Participants

Our study involved participants broadly categorized into two groups, e.g., Post-stroke (Grp ${}_{\mathrm {PS}}$) (M (SD) = 54 (15) Years) and Healthy (Grp ${}_{\mathrm {H}}$) Groups (M (SD) = 50 (15) Years) who were age-matched (p-value > 0.05).

All stroke survivors were in the chronic phase. The Grp_PS_ was further sub-divided as Aphasic (Grp ${}_{\mathrm {AP}}$) (M (SD) = 52 (17)) and Non-Aphasic (Grp ${}_{\mathrm {NAP}}$) Groups (M (SD) = 59 (11)) using WAB scores cutoff (AQ ${}^{\mathrm {O}} < 93.8=$ Aphasic [11]) (Table 1), who were again age-matched (p-value> 0.05). Only native Hindi-speaking individuals with school-leaving certification, and who did not face difficulties in using their hands while responding, were included. The post-stroke participants were recruited from hospitals in Delhi and Gandhinagar using convenience sampling. The recruitment process was facilitated by a team of doctors. The healthy participants were recruited from the neighbourhood. For each participant, we had access to Western Aphasia Battery (WAB) [11] scores. Our study followed Institute Ethics (IITGN/IEC/2024-25/26 and IEC-364/06.05.2022, RP-37/2022).

**TABLE 1:** Participants’ Characteristics.

	ID	Age (Sex)	AQ^O^	WAB (AQ ${}^{\mathrm {A}}$)	WAB (AQ ${}^{\mathrm {V}}$)	ID	Age (Sex)	AQ^O^	WAB (AQ ${}^{\mathrm {A}}$)	WAB (AQ ${}^{\mathrm {V}}$)
Aphasic Group (Grp ${}_{\mathrm {AP}}$)	S1	24(M)	61.6	45.6	81.5	H1	65(M)	97	98.3	86.75
	S2	30(F)	55.8	41.35	89.9	H2	65(M)	96.6	99.9	89.9
	S3	74(M)	80	43.1	80.3	H3	51(M)	93.6	88	100
	S4	40(M)	26.6	33.55	51.8	H4	39(M)	96	88.1	100
	S5	52(M)	78.3	27.05	78.6	H5	57(F)	100	88	100
	S6	28(M)	89.1	44.75	88.7	H6	44(M)	100	87.8	100
	S7	32(F)	40	42.25	91.2	H7	53(M)	100	87.9	100
	S8	33(F)	61.6	50	51.8	H8	40(M)	93.6	88	100
	S9	55(M)	49.4	3.65	10.5	H9	65(M)	96	87.8	100
	S10	69(M)	45	5.4	12.9	H10	40(M)	93.6	87.5	100
	S11	65(M)	78	5.4	28.7	H11	44(F)	94	88	100
	S12	58(M)	71	28.8	91.6	H12	71(M)	95	87.4	100
	S13	74(M)	72	35.2	87.1	H13	46(M)	99.2	87.8	100
	S14	75(M)	76	32.9	71.9	H14	78(M)	98.5	88.2	100
	S15	65(M)	36	29.25	78.2	H15	37(M)	99	87.2	100
Non-Aphasic Group (Grp ${}_{\mathrm {NAP}}$)	S16	40(M)	94.5	87.75	73.2	H16	83(M)	100	87.6	100
	S17	70(M)	100	26.15	74	H17	57(M)	98.8	88.1	100
	S18	71(M)	100	53.7	89	H18	65(M)	99.5	88.2	100
	S19	44(M)	100	43.45	79.8	H19	56(F)	99.1	88	100
	S20	51(M)	95	42.45	79.1	H20	44(M)	98.3	87.8	100
	S21	70(M)	96	43.45	89	H21	56(M)	97.9	88	100
	S22	54(M)	94	52.65	91.7	H22	35(M)	98.7	88.1	100
	S23	65(M)	96	46.7	93.2	H23	32(M)	98.4	88	100
	S24	54(M)	98	53.65	83.1	H24	70(F)	99.6	88.2	100
	S25	67(M)	98	38.1	87.8	H25	31(M)	98.2	88.1	100

Note: S1-S25: Post-stroke Group (Grp ${}_{\mathrm {PS}}$), H1-H25: Group Healthy (Grp ${}_{\mathrm {H}}$), AQ^A^ and AQ^V^: WAB scores (%) of sections relevant to Acoustic, and Visual and Reading tasks of CHASP, respectively, AQ^O^: Comprehensive AQ Score (%) of WAB, WAB: Western Aphasia Battery, CHASP: Computerized Hindi Aphasia Screening Platform.

#### 2) Experimental Setup

The experimental setup of the CHASP consisted of (i) two Computers, (ii) a Sensored Base Station, (iii) a Microcontroller-based event marker unit, and (iv) Physiology-based Data Acquisition Modules.

One computer (Task Computer meant for the participant) was used to present the various tasks (Section II-B.5) and acquire one’s response while computing one’s performance and logging the data in the backend. The other computer (meant for the experimenter) displayed a replica of the participant’s screen to help the experimenter to observe and assist the participant, if needed. Both computers had touch-sensitive screens and were oriented at 90° to one another to replicate a conventional clinician-patient setup [26] (Fig. 4(a)).

**FIGURE 4.**
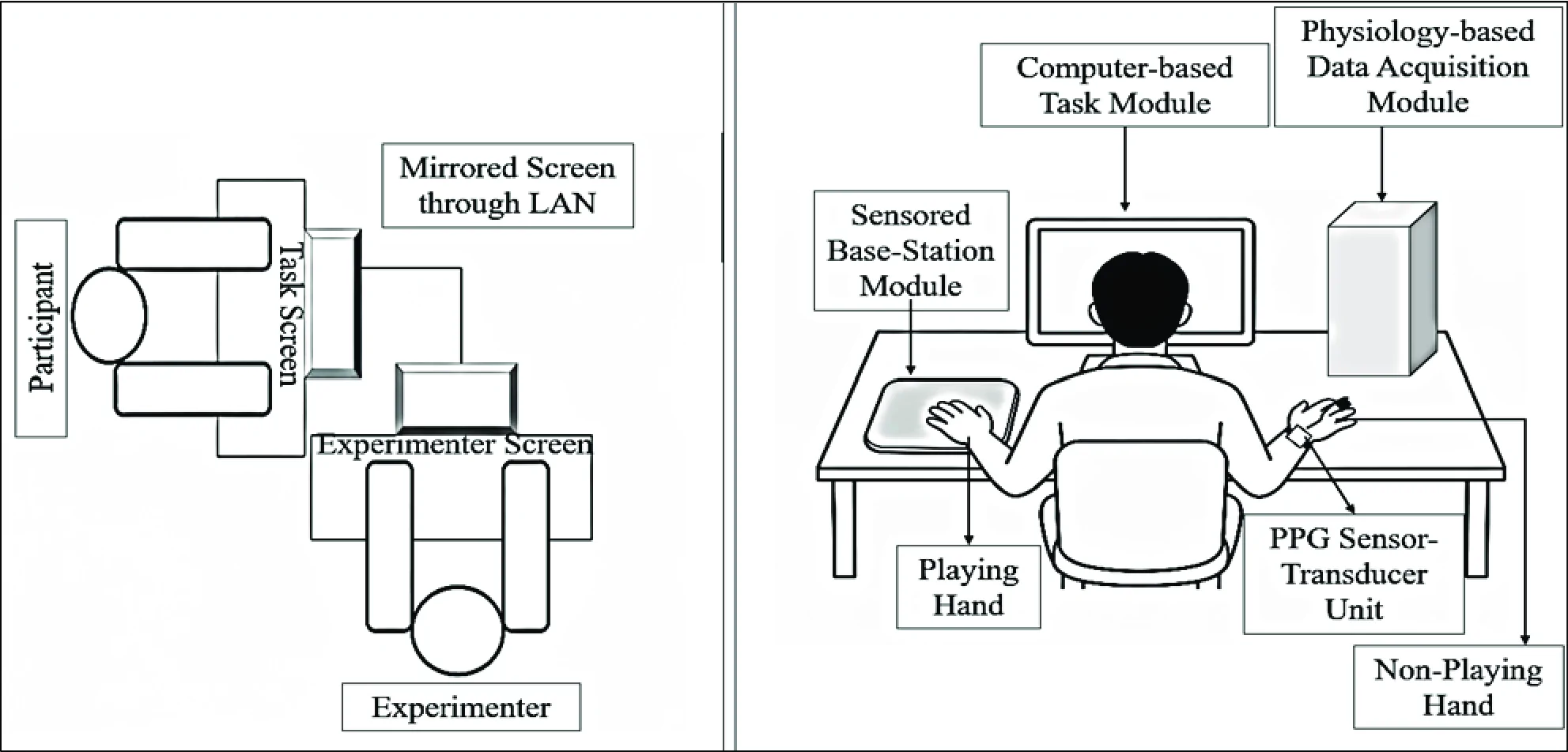
Experiment Setup showing the (a) seating arrangement of the participant and experimenter, and (b) Data Acquisition setup.

The sensor-enabled Base Station was integrated with the Task Computer via a microcontroller-enabled interface unit for storing the time-stamped hand-status information. The Microcontroller-based event marker unit connected the USB port of the Task Computer with the Digital Input/Output card of the Biopac MP150 module. This module housed the receiver unit that was interfaced with the transmitter unit connecting a PPG sensor mounted on a Velcro belt.

#### 3) Procedure

Our study required a commitment of approximately 20 minutes from each participant, during which one interacted with the computer-based tasks. Upon entering the study room, the participant was asked by the experimenter to sit comfortably on a chair. Then, the experimental setup along with the tasks were explained to the participant. Also, one was free to withdraw from the study if uncomfortable and can ask for breaks, if required. Signing of the consent form was administered once the participant expressed that he/she has understood what was expected from him / her and was ready to take part in the study. Then, the participant’s demographics were collected. This was followed by asking the participant to indicate his / her hand which he / she plans to use for interacting with our task (playing hand henceforth with the other hand being considered as the non-playing hand). The participant was assisted in wearing the Velcro belt carrying a light-weight wireless transmitter on the wrist and a velcro strip carrying a PPG sensor on the distal phalange of the middle finger of the non-playing hand. Also, the participant was asked to place the palm of his / her playing hand on the Sensored Base Station (Fig. 4) to start with and after responding to each sub-task. Subsequently, the experimenter started the task execution. Each task began with a screen delivering instruction in audio-visual mode. This was followed by a sub-task belonging to any of the Acoustic, or Visual and Reading task types. Following this, the participant was expected to respond to the sub-task by lifting his / her playing hand from the Sensored Base Station followed by either *screen touch* or *drag-and-drop* based on the sub-task. Once the tasks were completed, the experimenter thanked the participant.

#### 4) Computation of Performance Score

Upon completion of a sub-task, based on each correct and incorrect response being awarded a score of *‘1’,* or *‘0’*, respectively, the CHASP was used to compute one’s performance score in terms of % performance score which in turn was normalized on a 0 to 1 scale.

\begin{equation*} Normalized~Score_{ij}=\frac {Score{\,}ij\mathrm {- }min{\,}(Scorej)}{max(Scorej\mathrm {) - }min(Scorej)} \tag {1}\end{equation*}

where *i* *(= 1 to 50)* denotes participant id, *j* *(= 1 to 10)* the task, min (*Score* ${}_{j}$), the minimum and max (*Score* ${}_{j}$), the maximum Performance Score across participants and tasks.

#### 5) Computation of Normalized Reaction Time

After the presentation of a sub-task, the CHASP computed the Response Time based on the time-stamped hand-status information captured using the sensored Base Station (Section II.*2*). The Response Time (in seconds) was normalized (0 to 1 scale) using Min-Max normalization [27] for the Acoustic, and Visual and Reading task types using Eq. (2).

\begin{equation*} Normalized~{RT}_{ij}=\frac {RTij-min\mathrm { (}RTij)}{max(RTij\mathrm {) - }min(RTij)} \tag {2}\end{equation*}

where *RT* ${}_{ij}$ is the Response Time of the $i^{th}$ (= 1 *to 50)* participant for the $j^{th}$*(= 1 to 5)* for Acoustic sub-task and *(= 1 to 2)* for Visual and Reading sub-task, min(*RT* ${}_{ij}$) and max(*RT* ${}_{ij}$) are the minimum and maximum Response Time for all participants for each of the tasks, respectively.

#### 6) Computation of Normalized Interbeat Interval

The CHASP was used to capture the PPG signal corresponding to the interval between the presentation of a sub-task and the instant of one’s response as marked by sensor-enabled Base Station based on hand-status (Section II-A.2. The PPG signal was acquired using the BIOPAC MP150 at a sampling rate of 1000 samples/sec and logged at the backend of the Task Computer executing the data acquisition *AcqKnowledge* software (that comes with BIOPAC MP150). Simultaneously, the recorded signals were event-marked based on transition of sub-tasks. Subsequently, the acquired signal was processed offline for extracting features for each sub-task. For this, the acquired signal was filtered using a second-order Butterworth band-pass filter with cut-off frequencies of 0.5 and 8 Hz [28] using the *sosfiltfilt* function from SciPy signal-processing library of Python. This procedure suppressed baseline drift and high-frequency noise without introducing phase displacement. In addition, we took care of motion artefacts by minimizing such possibilities by asking each participant to place their non-playing hand (having the PPG sensor) on the table. Also, we analyzed the PPG signals acquired between the instants of task presentation and the hand-status data indicating a hand lift (Section II-A2). Then, peaks in the signal were detected using the *find peaks* function of the *SciPy* library, with valid successive peaks being the ones having a minimum separation *≥ 0.5* s [28]. For obtaining the IBI, we first computed one’s Heart Rate (HR) for each sub-task from the detected peaks. For example, for participant *i* and sub-task *j*, the HR for a sub-task was calculated from the number of detected peaks and the duration of the filtered PPG signal segment using Eq. (3).

\begin{equation*} {HR}_{ij}=\frac {N_{ij}}{T_{ij}}\times 60 \tag {3}\end{equation*}

Here, $N_{ij}$ represents the number of detected valid peaks and $T_{ij}$ represents the analyzed segment duration in seconds. The IBI was subsequently derived using Eq. (4).

\begin{equation*} {IBI}_{ij}=\frac {60}{{HR}_{ij}}\mathrm {= }\frac {T_{ij}}{N_{ij}} \tag {4}\end{equation*}

Finally, the IBI was normalized (0 to 1 scale) with Min–Max normalization [27] using Eq. (5).

\begin{equation*} Normalized~{IBI}_{ij}=\frac {IBIij-min~\mathrm { (}IBIij)}{max(IBIij\mathrm {) - }min(IBIij)} \tag {5}\end{equation*}

where *IBI* ${}_{ij}$ is the Interbeat Interval of the $i^{th}$ (= 1 *to 50)* participant for the $j^{th}$*(= 1 to 5)* for Acoustic sub-task and *(= 1 to 2)* for Visual and Reading sub-task. Again, min (*IBI* ${}_{ij}$) and max (*IBI* ${}_{ij}$) are the minimum and maximum values of IBI across all participants for each of the tasks, respectively.

#### 7) Scoring Using Western Aphasia Battery

The Western Aphasia Battery (WAB) is considered as one of the gold-standard clinical tools for assessing Aphasia [11] based on one’s performance in a task. The WAB comprises 8 tasks namely, (i) Spontaneous Speech (Task ${}_{\mathrm {WAB1}}$) having sub-tasks e.g., ‘*Conversational Questions*’ and ‘*Picture Description*’, (ii) Auditory Verbal Comprehension (Task ${}_{\mathrm {WAB2}}$) having sub-sections e.g., Yes/No, Auditory Word Recognition and ‘*Sequential Commands*’), (iii) Repetition (Task ${}_{\mathrm {WAB3}}$), (iv) Naming and Word Finding (Task ${}_{\mathrm {WAB4}}$) having sub-sections e.g., Object Naming, Word Fluency, Sentence Completion, Responsive Speech, (v) Reading (Task ${}_{\mathrm {WAB5}}$) having sub-sections e.g., Comprehension of Sentences, Reading Commands, Written Word–Object Choice Matching, Written Word Picture Choice Matching, Picture-written word choice matching, Spoken word–written word choice matching, Letter discrimination, Spelled word recognition and Spelling, (vi) Writing (Task ${}_{\mathrm {WAB6}}$) having sub-sections e.g., Writing Upon Request, Written Output, Writing to Dictation, Writing Dictated Words, Dictated Letters and Numbers, Alphabet and Numbers, and Copying sentence, (vii) Apraxia (Task ${}_{\mathrm {WAB7}}$), and (viii) Constructional, Visuospatial and Calculation (Task ${}_{\mathrm {WAB8}}$) having sub-sections e.g., Drawing, Block Design, Calculation and Raven’s Colored Progressive Metrices. While the scoring was computed for each of the Acoustic, and Visual and Reading tasks (Section II-A.1) of CHASP, we wanted to understand the clinical significance of the CHASP-based scores in terms of their corroboration with the WAB measures. However, with the CHASP offering task types having a specific set of sub-tasks, we wanted to consider the tasks and sub-tasks of the WAB that are relevant to those offered by the CHASP.

Specifically, we considered ‘*Sequential Commands*’ sub-task under the Task_WAB2_, ‘*Dictated Letters and Numbers*’ sub-task under the Task_WAB5_ and Task_WAB7_ to compute the AQ^A^ (Table 1) in line with the five sub-tasks of the Acoustic task offered by the CHASP (Section II-A.1.a). Again, we considered ‘*Picture-written word choice matching*’ sub-task and ‘*Comprehension of Sentences*’ sub-task under the Task_WAB5_ to compute the AQ^V^ (Table 1) in line with the two sub-tasks of the Visual and Reading task (Section II.1.*1.3*) offered by CHASP. The CHASP scores were obtained for each individual participating in the study.

#### 8) Clinical Relevance Using Pearson’s Correlation

To understand the clinical significance of CHASP-based scores in terms of their corroboration with the scores of the relevant sections of WAB (Section II-B.7), we computed the Pearson’s Correlation [25] between these measures (Eq. (6)).

\begin{equation*} \frac {\sum \nolimits _{i=1}^{N} (x_{i}-\bar {x})(y_{i}-\bar {y})}{\sqrt {\sum \nolimits _{i=1}^{N} (x_{i}-\bar {x})^{2}} \sqrt {\sum \nolimits _{i=1}^{N} (y_{i}-\bar {y})^{2}}} \tag {6}\end{equation*}

#### 9) Statistical Analysis

Here statistical analyses were carried out using Statistical Package for the Social Sciences (SPSS Statistics; Version 20). First, an ANOVA test [29] was conducted on the Performance Score, Response Time, and Interbeat Interval. Then, residuals were tested for normality using Shapiro-Wilk test [29]. As the distribution was found to be non-parametric, between-group differences were assessed using Mann–Whitney U test [29] and within-group assessment was done using Wilcoxon Signed-Rank test [29]. Also, effect sizes were considered alongside p-values to describe the magnitude of group differences among the participants. Specifically, *Cohen’s* *d* *value* [30] was computed in each case.

## III. Results and Discussion

Here, we conducted a study involving a group of post-stroke participants (Grp_PS_ having Aphasic (Grp ${}_{\mathrm {AP}}$) and non-aphasic (Grp ${}_{\mathrm {NAP}}$)) individuals, and a group of age-matched healthy counterparts (Grp ${}_{\mathrm {H}}$). The participants interacted with the CHASP (Section II-A) offering Acoustic, and Visual and Reading Tasks while we computed their Performance scores (Section II-A), and Response time (Section II-A), and Interbeat interval (IBI, a physiological biomarker; Section II-A) for each task. Also, we had access to their WAB scores. We aimed to understand the (i) potential of the two types of tasks of CHASP in differentiating (a) Post-stroke and Healthy Groups and (b) Aphasic and Non-Aphasic groups in terms of performance and IBI, and (ii) clinical significance of the two types of tasks in terms of its corroboration with the scores in specific tasks and sub-tasks of the WAB that are in line with those offered by the CHASP (Table 1).

### A. Understanding the Potential of the Computerized Platform in Differentiating Post-Stroke and Healthy Groups

We wanted to understand the potential of the tasks offered by CHASP (Section II) to differentiate Grp_PS_ and Grp_H_ in terms of (i) performance measures, e.g., (a) Performance scores and (b) Response Time, and (ii) Interbeat Interval.

#### 1) Differentiating in Terms of Performance Scores

One’s quality of task execution can be quantified in terms of performance score. Thus, we analyzed the performance scores of Grp_H_ and Grp_PS_ in the various tasks. From Fig. 5(a), we can see that Grp_PS_ had a lower % Correct response than the Grp_H_ irrespective of the type of task (with statistical difference, p-value < 0.01, Cohen’s d = 1.5 for Acoustic and p-value < 0.05, Cohen’s d = 0.9 for Visual and Reading tasks). Again, for Grp_H_, the % Correct response in the Visual and Reading task was statistically higher ( *Δ =12*% on average, p-value < 0.05, Cohen’s d = 0.934) than the Acoustic tasks. Similarly, for Grp_PS_, the % Correct response in the Visual and Reading task was statistically higher ( *Δ =32.5*% on an average with p-value < 0.01, Cohen’s d = 1.46) than the Acoustic tasks.

**FIGURE 5.**
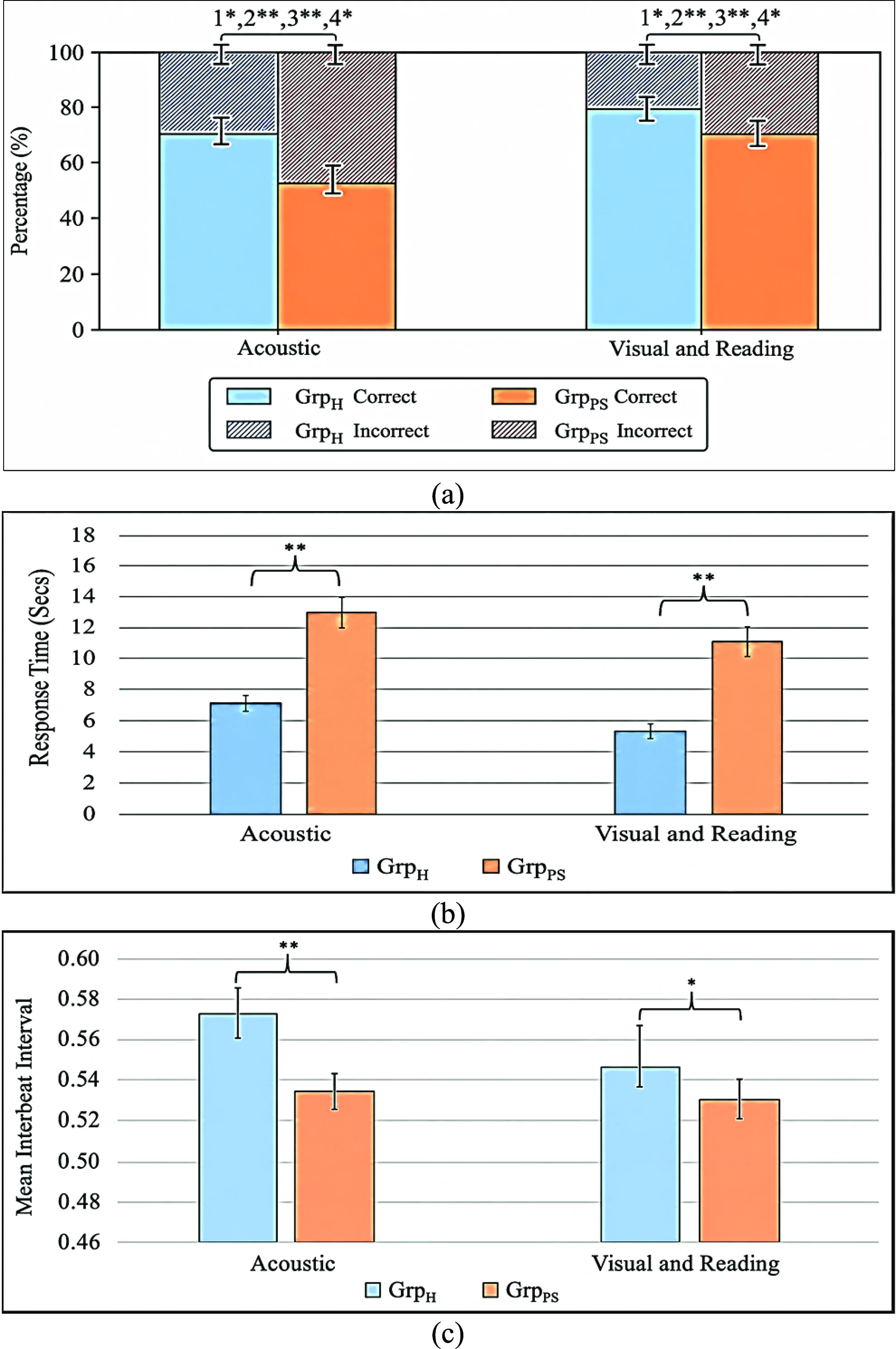
(a) Percentage of correct and incorrect responses, (b) Response Time, and (c) Mean Interbeat Interval (Mean IBI) of Grp_PS_ and Grp_H_ while performing Acoustic, and Visual & Reading tasks. Note- ${}^{\ast }$: p < 0.05, ${}^{\ast \ast }$: p < 0.01, 1: Statistical difference between correct responses of (Grp_PS_) and (Grp_H_), 2: Statistical difference between incorrect responses of (Grp_PS_) and (Grp_H_), 3: Statistical difference between correct responses and incorrect responses among (Grp_H_), and 4: Statistical difference between correct responses and incorrect responses among (Grp_PS_); GrpPS: Post-stroke Group, Grp_H_: Healthy Groups.

To summarize, among the two types of tasks offered by CHASP, the Acoustic tasks seemed to be comparatively more difficult than the Visual and Reading task irrespective of the participant group. Also, the Grp_PS_ performed worse than the Grp_H_ irrespective of the type of tasks. This observation can be possibly attributed to the deficits of post-stroke individuals in semantic integration, multisensory binding and executive functioning, adversely affecting their response accuracy across language, visual processing, and auditory cue-based decision tasks [31].

#### 2) Differentiating in Terms of Response Time

Apart from judging the quality of one’s performance in a task in terms of scores, it is also important to understand the time one took while deciding on a response, i.e., Response Time (Section II-A), since this duration might be indicative of the underlying cognitive processing involved in making a decision [29]. It can be seen from Fig. 5 (b) that the Response Time was significantly (p-value < 0.01, Cohen’s d = 1.6 for Acoustic and Cohen’s d = 1.5 for Visual and Reading) higher for Grp_PS_ than Grp_H_, irrespective of the type of task ( *Δ =45*% and 53% for Acoustic, and Visual and Reading task, respectively). Such an observation can be attributed to post-stroke individuals often demonstrating reduced processing speed than the healthy [27]. Again, we can see that Grp_H_ demonstrated longer Response Time for the Acoustic tasks compared to the Visual and Reading tasks (the Response Time for Acoustic module being statistically different (p-value < 0.05, Cohen’s d = 0.934) from Visual and Reading tasks). In contrast, for Grp_PS_, comparable Response time was observed for the Acoustic task, and the Visual and Reading task (p-value > 0.05).

To summarize, the Grp_PS_ took more time (on an average) to respond than the Grp_H_ irrespective of the two types of tasks, with the Response Time for Grp_PS_ being the highest for the Acoustic task. Such an observation can be possibly attributed to the audio comprehension, lexical and semantic processing required in executing Acoustic tasks that are often challenging for post-stroke individuals [31].

#### 3) Differentiating in Terms of Biomarker

Having seen that the Grp_PS_ took more time to initiate a response and performed worse than Grp_H_ irrespective of the type of task, possibly inferring that the tasks were more difficult for Grp_PS_ than the Grp_H_, we wanted to see whether the felt difficulty can be quantified in terms of a biomarker. For this, we chose the mean Interbeat Interval (IBI) which is an indicator of felt difficulty [31] and can be easily measured in a non-invasive manner [Section II-A.3]. From Fig. 5(c), it can be seen that irrespective of the type of task, the Grp_PS_ demonstrated statistically lower mean IBI than the Grp_H_ (p-value < 0.01, Cohen’s d = 0.7 for each of Acoustic and p-value < 0.05, Cohen’s d = 0.4 for the Visual and Reading tasks) possibly inferring higher difficulty [31] felt by Grp_PS_ than the Grp_H_. Again, we can see that for the Grp_H_, the Mean IBI was lower for the Visual and Reading task compared to the Acoustic tasks. In contrast, for the Grp_PS_, the Mean IBI was comparable for the Acoustic and Visual and Reading tasks. However, while comparing Mean IBI across the types of tasks within each participant group, we could not find any statistical difference. Thus, though there existed statistical differences in the felt difficulty among participant groups irrespective of the type of tasks, yet within each participant group, the extent of felt difficulty might have been comparable across types of tasks.

Having seen that irrespective of the two types of tasks, the indices, namely Mean IBI, % Correct Response (Performance score) and Response Time (Section III-B) were potent in differentiating the Grp_PS_ and the Grp_H_, we wanted to understand whether both the tasks and the indices are equally potent to distinguish the Aphasic and Non-Aphasic groups (Grp_AP_ and Grp_NAP_, respectively, Table 1).

### B. Understanding the Potential of the Computerized Platform in Differentiating Aphasic and Non-Aphasic Groups

For this, we analyzed the responses of the Aphasic group (Grp ${}_{\mathrm {AP}}$) and Non-Aphasic group (Grp ${}_{\mathrm {NAP}}$) while they interacted with the Acoustic and Visual and Reading tasks offered by the CHASP (Section II-B.1).

#### 1) Differentiating in Terms of Performance Scores

We analyzed the performance scores of Grp_AP_ and Grp_NAP_ for the Acoustic, and Visual and Reading tasks. From Fig. 6(a), we can see that for the Acoustic task, Grp_AP_ demonstrated a statistically lower percentage of correct responses than Grp_NAP_ (p-value < 0.01, Cohen’s d = 1.4), indicating that the Acoustic task was sensitive in differentiating Aphasic and Non-Aphasic post-stroke individuals. Such an observation might possibly be attributed to the fact that Acoustic tasks rely on auditory processing, comprehension, phonological working memory, and response selection, which are often affected in individuals with Aphasia [31].

**FIGURE 6.**
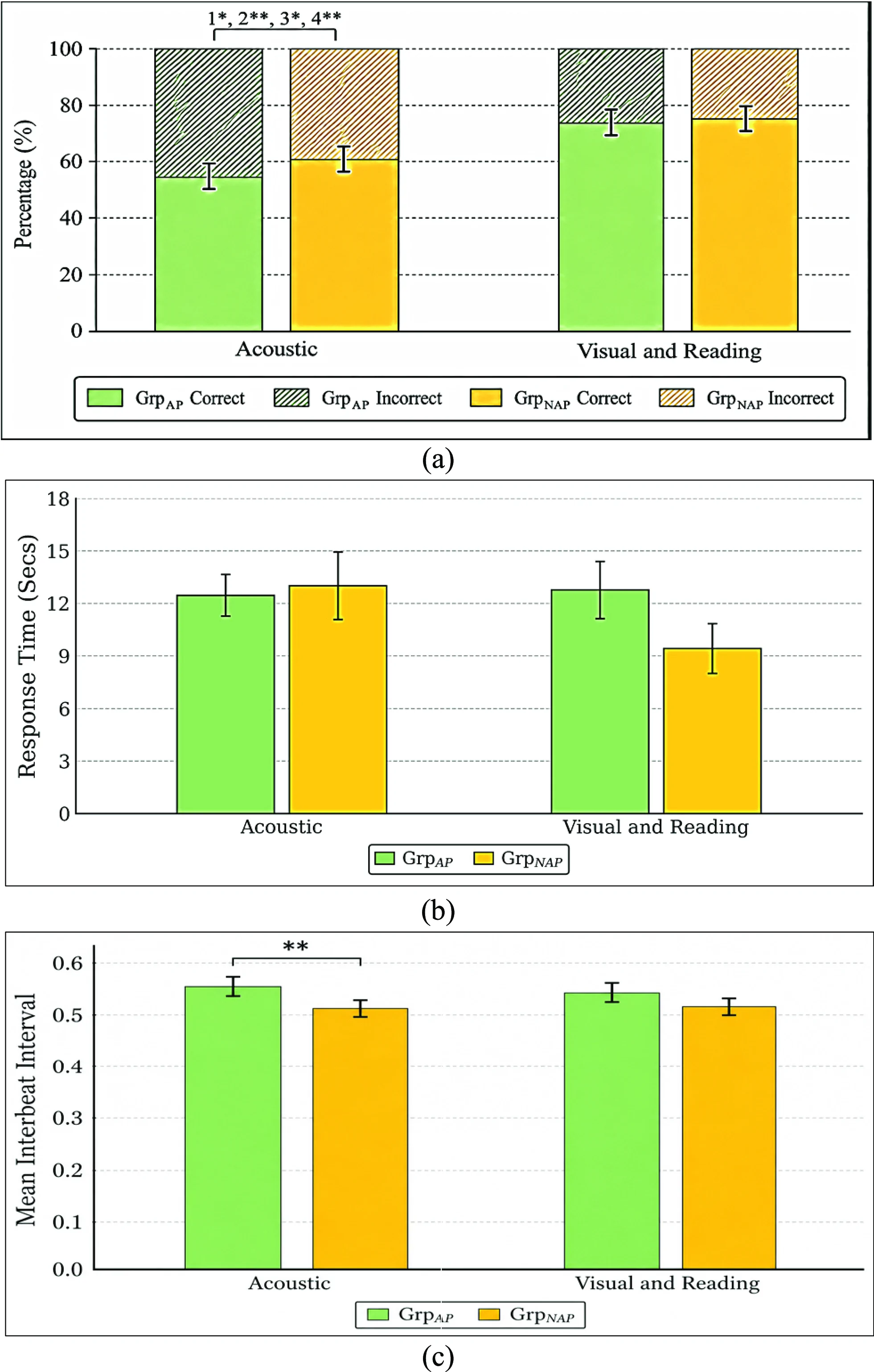
(a) Percentage of correct and incorrect responses, (b) Response time, and (c) Mean interbeat interval (Mean IBI) of Grp_AP_ and Grp_NAP_ while performing acoustic, and visual & reading tasks. Note- Mean IBI $= ^{\ast } $: p-value ¡ 0.05, ${}^{\ast \ast }$: p-value < 0.01, Grp ${}_{\text {AP}} =$ Aphasic and Grp ${}_{\text {NAP}} =$ Non-Aphasic Groups. 1: Statistical difference between correct responses of (Grp_AP_) and (Grp_NAP_), 2: Statistical difference between incorrect responses of (Grp_AP_) and (Grp_NAP_), 3: Statistical difference between correct responses and incorrect responses among (Grp_AP_), and 4: Statistical difference between correct responses and incorrect responses among (Grp_NAP_)

In contrast, for the Visual and Reading task, the performance scores of Grp_AP_ and Grp_NAP_ were comparable, with no statistically significant difference between the two groups (p-value > 0.05). This may indicate that, in the present participant pool, the Visual and Reading task was less sensitive than the Acoustic task in differentiating Aphasic and Non-Aphasic post-stroke individuals.

To summarize, among the two types of tasks offered by CHASP, the Acoustic task showed stronger potential than the Visual and Reading task in differentiating Grp_AP_ and Grp_NAP_ in terms of % Correct response possibly inferring that tasks relying on auditory processing, comprehension and phonological working memory impose cognitive burden on Aphasic individuals [33].

#### 2) Differentiating in Terms of Response Time

In addition to evaluating task performance through scores, it is important to examine the duration required by a participant to decide and initiate a response, i.e., Response Time, since this duration might infer the underlying cognitive processes involved in making a decision [34]. From Fig. 6(b), we can see that Grp_AP_ and Grp_NAP_ demonstrated comparable Response time with no statistical difference (p-value > 0.05) in both the Acoustic and Visual and Reading tasks. Such an observation might possibly infer comparable post-stroke-related slowing in processing speed and task execution in the two participant groups.

To summarize, though the Response Time could statistically differentiate the Post-stroke and Healthy groups, yet the Response Time was not potent to differentiate the two post-stroke sub-groups, i.e., the Aphasic and Non-Aphasic groups.

#### 3) Differentiating in Terms of Biomarker

Having observed the performance-based differences between Grp_AP_ and Grp_NAP_, particularly for the Acoustic task, we further wanted to understand whether this difference could also be captured through a physiology-linked biomarker. For this, we considered the mean Interbeat Interval (Mean IBI), which can be used as an indicator of task-related felt difficulty [22].

From Fig. 6(c), we can see that for the Acoustic task, the Grp_AP_ demonstrated statistically lower mean IBI than Grp_NAP_ (p-value < 0.01, Cohen’s d = 1.1). This may indicate greater task-related physiological load or felt difficulty in Grp_AP_ than the Grp_NAP_ while performing Acoustic tasks. This observation is also in line with the performance-score findings, where Grp_AP_ showed significantly lower accuracy than Grp_NAP_ in the Acoustic task. In contrast, for the Visual and Reading task, the mean IBI values of Grp_AP_ and Grp_NAP_ were comparable, with no statistically significant (p-value > 0.05) difference between the two groups while echoing the observations on the performance-score for the Visual and Reading task (Fig. 6(c)).

To summarize, the Mean IBI was found to be potent in differentiating the Grp_AP_ and Grp_NAP_ specifically for the Acoustic tasks. Such observations led us to dig deeper to understand whether these CHASP-derived measures corresponding to the Acoustic, and Visual and Reading tasks were clinically relevant.

### C. Understanding the Clinical Significance of the Computerized Platform

With regard to screening of post-stroke Aphasic individuals, we could see the importance of two of the CHASP-derived measures, namely Performance Score and Mean IBI.

This raises an important question regarding the clinical relevance of these CHASP measures. Given that one’s task-based score is a widely accepted measure, particularly when it comes to the use of currently-existing tools for assessing Aphasia, we explored the Performance score corresponding to the Acoustic and Visual and Reading tasks derived from CHASP and correlated (using Pearson’s Correlation (Section II-B.8)) these with the AQ^A^ and AQ^V^ scores (Table 1) corresponding to the sections of WAB (to which we had access) that were relevant to the tasks offered by CHASP.

From Fig. 7, we can see that the group average % Correct responses (considered after averaging participant level scores) of Grp_AP_ and Grp_NAP_ in the Acoustic, and Visual and Reading tasks offered by the CHASP strongly correlated (r *≥ 0.7* [35]) with the participant level performance score in the specific tasks and sub-tasks of the WAB that are in line with those of the CHASP. Such an observation possibly infers the importance of checking one’s comprehension ability (oral or visual) and the potential of CHASP to serve as a complementary screening tool that can have clinical relevance.

**FIGURE 7.**
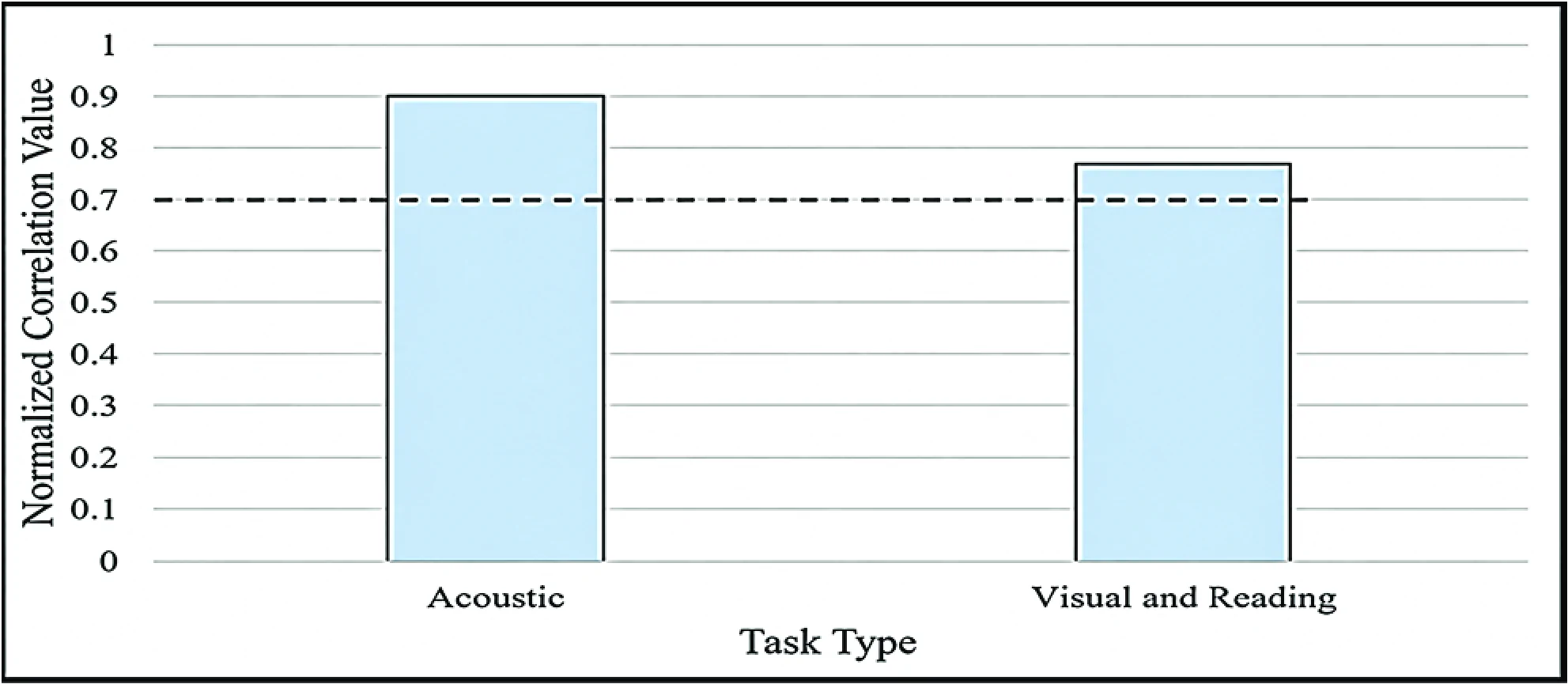
Correlation of scores in CHASP and relevant sections WAB. Note- WAB: Western Aphasia Battery, CHASP: Computerized Hindi Aphasia Screening Platform.

## IV. Conclusion

In the present work, we have developed a Computerized Hindi Aphasia Screening Platform (CHASP) as a physiology-sensitive computer-based digital screening tool offering two types of tasks, namely Acoustic, and Visual and Reading tasks. While our post-stroke group (further categorized into Aphasic and Non-Aphasic groups) and their age-matched healthy counterparts interacted with the CHASP, we acquired their performance accuracy in terms of % correct response, response time, and physiological response (interbeat interval; IBI). Results of our study showed that both the tasks were potent in differentiating the post-stroke and healthy groups with the post-stroke participants consistently having lower performance scores, prolonged response times, and reduced IBI inferring adversely affected response accuracy across language, visual processing, and auditory cue-based decision tasks, reduced processing speed and increased felt difficulty than their healthy counterparts. In contrast, for the Aphasic and Non-Aphasic sub-groups, our results showed that both the CHASP-derived measures, namely performance scores and IBI could differentiate the two sub-groups, particularly for the Acoustic task. Also, our results showed the importance of checking one’s comprehension ability (oral or visual) and the potential of CHASP to serve as a clinically-relevant screening tool.

Though our results are promising, yet certain limitations exist. Given that our study was an exploratory one, we recruited limited number of Aphasic and Non-Aphasic participants, though summing up to 25 numbers of participants in each of the post-stroke and healthy groups with an overall sample strength of 0.8 based on G-Power Statistic [36]). However, in future, we plan to recruit larger participant pool for clinical studies. Also, in our present study, majority of our participants were male. In future studies, we would like to include a balanced pool of male and female participants. Though there exist different assessment modalities, such as International Classification of Functioning (ICF), use of pen-and-paper based tools, etc., the current platform was adopted from the Indian Aphasia Battery which is commonly being practiced by the clinicians in India with the language being Hindi and targeted for native-Hindi speakers. With India being multi-lingual country, in future, the CHASP can be extended to other Indian languages. Also, in future we will ensure that the loudness of the speaker (i.e., the therapist) collecting one’s responses to pen-and-paper based questions is adequate for the respondents to hear the questions being asked. Again, in our present work, the CHASP is augmented with Pulse Plethysmogram to facilitate the acquisition of the Interbeat Interval. In future, we plan to add other biomarkers while tapping one’s looking pattern, electrodermal activity, etc. which might offer additional insights into one’s cognitive state while deciding on a response.

Notwithstanding the limitations, our present study offers a digitized screening platform in Hindi language, thereby laying the foundation stone towards a more accessible screening tool particularly for native-Hindi speakers that can move beyond clinical settings and serve as a complementary tool in the hands of clinicians working with this target group.

## Acknowledgment

The authors would like to thank Dr. Jashrit Dubey (Physiotherapist, Narayan Physio Care) and Dr. Akshar Naik (Speech Therapist, Swar Speech and Swallowing Rehabilitation Center) along with all the participants for their cooperation and valuable time. In addition, they would like to thank BIRAC, DBT, SERB, and Tata Innovation Fellowship for their support. Ashima Nehra and Uttama Lahiri are joint last authors. Ashima Nehra is responsible for clinical inputs. Uttama Lahiri is responsible for technical inputs, data analysis, and project implementation.

## Conflict of Interest Statement

The authors do not have any conflict of interest to declare. They disclose any financial and personal relationships with other people or organizations that could inappropriately influence (bias) their work.

## References

[1] H. Goodglass and E. Kaplan, Assessment of Aphasia and Related Disorders, 2nd ed., Philadelphia, PA, USA: Lea & Febiger, 1983.

[2] M. L. Berthier, “Poststroke aphasia: Epidemiology, pathophysiology and treatment,” Drugs Aging, vol. 22, no. 2, pp. 163–182, 2005. 10.2165/00002512-200522020-00006 15733022

[3] A. Croquelois and J. Bogousslavsky, “Stroke aphasia: 1,500 consecutive cases,” Cerebrovascular Diseases, vol. 31, no. 4, pp. 392–399, 2011, doi: 10.1159/000323217. 21346340

[4] S. Kamalakannan , “Incidence & prevalence of stroke in India: A systematic review,” Indian J. Med. Res., vol. 146, no. 2, pp. 175–185, 2017. 10.4103/ijmr.IJMR_516_15 PMC5761027 29265018

[5] A. C. Laska, T. Kahan, A. Hellblom, V. Murray, and M. von Arbin, “A randomized controlled trial on very early speech and language therapy in acute stroke patients with aphasia,” Cerebrovascular Diseases Extra, vol. 1, no. 1, pp. 66–74, Jul. 2011. 10.1159/000329835 PMC3343759 22566984

[6] B. Mohr, “Neuroplasticity and functional recovery after intensive language therapy in chronic post stroke aphasia: Which factors are relevant?” Frontiers Human Neurosci., vol. 11, p. 332, Jun. 2017. 10.3389/fnhum.2017.00332 PMC5487528 28701937

[7] B. Crosson , “Functional MRI of language in aphasia: A review of the literature and the methodological challenges,” Neuropsychology Rev., vol. 17, no. 2, pp. 157–177, Jun. 2007. 10.1007/s11065-007-9024-z PMC2659355 17525865

[8] S. Murali , “Bringing MRI to low- and middle-income countries: Directions, challenges and potential solutions,” NMR Biomed., vol. 37, no. 7, p. 4992, 2024. 10.1002/nbm.4992 37401341

[9] A. Krishnan, M. Asadullah, R. Kumar, R. Amarchand, R. Bhatia, and A. Roy, “Prevalence and determinants of delays in care among premature deaths due to acute cardiac conditions and stroke in residents of a district in India,” Lancet Regional Health - Southeast Asia, vol. 15, Aug. 2023, Art. no. 100222. 10.1016/j.lansea.2023.100222 PMC10442961 37614354

[10] N. Simmons-Mackie and A. Kagan, “Application of the ICF in aphasia,” Seminars Speech Lang., vol. 28, no. 4, pp. 244–253, Nov. 2007, doi: 10.1055/s-2007-986521. 17935009

[11] A. Kertesz, “The western aphasia battery: A systematic review of research and clinical applications,” Aphasiology, vol. 36, no. 1, pp. 21–50, 2022.

[12] S. S. Mahmoud, R. F. Pallaud, A. Kumar, S. Faisal, Y. Wang, and Q. Fang, “A comparative investigation of automatic speech recognition platforms for aphasia assessment batteries,” Sensors, vol. 23, no. 2, p. 857, Jan. 2023, doi: 10.3390/s23020857. PMC9863375 36679654

[13] H. Kaur, S. Bajpai, D. Pershad, V. Sreenivas, and A. Nehra, “Development and standardization of Indian aphasia battery,” J. Ment. Health Hum. Behav., vol. 22, no. 2, pp. 116–122, 2017.

[14] A. Győrfi , “Current assessment practices in aphasia therapy with special focus on tests and protocols used by AphasiaBank contributors,” J. Commun. Develop. Stud., vols. V–VI, pp. 2–13, 2019.

[15] M. Premanath, “Beyond numbers: Enhancing healthcare quality in India,” APIK J. Intern. Med., vol. 5, pp. 243–248, Apr. 2025.

[16] M. V. Ivanova , “The Russian aphasia test: A comprehensive, quantitative, standardized, and computerized language battery,” PLoS ONE, vol. 16, no. 11, 2021, Art. no. 0258946. 10.1371/journal.pone.0258946 PMC8601577 34793469

[17] S. M. Wilson, D. K. Eriksson, S. M. Schneck, and J. M. Lucanie, “A quick aphasia battery for efficient, reliable, and multidimensional assessment of language function,” PLoS ONE, vol. 13, no. 2, Feb. 2018, Art. no. e0192773, doi: 10.1371/journal.pone.0192773. PMC5806902 29425241

[18] D. Howard, K. Swinburn, and G. Porter, “Putting the CAT out: What the comprehensive aphasia test has to offer,” Aphasiology, vol. 24, no. 1, pp. 56–74, Jan. 2010, doi: 10.1080/02687030802453202.

[19] Y. Inamoto , “A tablet-based aphasia assessment system ‘STELA’: Feasibility and validation study,” JMIR Formative Res., vol. 7, Feb. 2023, Art. no. e42219, doi: 10.2196/42219. PMC9947769 36753308

[20] Y. E. Guo , “Assessment of aphasia across the international classification of functioning, disability and health using an iPad-based application,” Telemedicine e-Health, vol. 23, no. 4, pp. 313–326, Apr. 2017. 10.1089/tmj.2016.0072 27802112

[21] A. J. Privitera, S. H. S. Ng, A. P.-H. Kong, and B. S. Weekes, “AI and aphasia in the digital age: A critical review,” Brain Sci., vol. 14, no. 4, p. 383, Apr. 2024, doi: 10.3390/brainsci14040383. PMC11047933 38672032

[22] L. J. M. Mulder, “Measurement and analysis methods of heart rate and respiration for use in applied environments,” Biol. Psychol., vol. 34, nos. 2–3, pp. 205–236, Nov. 1992. 10.1016/0301-0511(92)90016-n 1467394

[23] C-16 Population By Mother Tongue, Census of India 2011, Ministry of Home Affairs, Government of India, New Delhi, India, 2018.

[24] Interlink Electronics, Camarillo, CA, USA. (2022). FSR ® Force Sensing Resistor Integration Guide. [Online]. Available: https://www.interlinkelectronics.com/fsr

[25] BIOPAC Systems, Inc., Goleta, CA, USA. (2022). MP150 Data Acquisition System-Hardware Manual. [Online]. Available: https://www.biopac.com/product/mp150-data-acquisition-system/

[26] J. E. Theodoros, R. Russell, C. Ward, and M. Wootton, “In-person versus telehealth assessment of discourse ability in adults with traumatic brain injury,” Brain Impair., vol. 10, no. 3, pp. 226–236, 2009.

[27] T. Hastie, R. Tibshirani, and J. Friedman, The Elements of Statistical Learning, 2nd ed., New York, NY, USA: Springer, 2009.

[28] M. Elgendi, I. Norton, M. Brearley, D. Abbott, and D. Schuurmans, “Systolic peak detection in acceleration photoplethysmograms measured from emergency responders in tropical conditions,” PLoS ONE, vol. 8, no. 10, Oct. 2013, Art. no. e76585, doi: 10.1371/journal.pone.0076585. PMC3805543 24167546

[29] J. H. Zar, Biostatistical Analysis, 5th ed., Upper Saddle River, NJ, USA: Prentice-Hall, 2010.

[30] J. Cohen, Statistical Power Analysis for the Behavioral Sciences, 2nd ed., Hillsdale, NJ, USA: Lawrence Erlbaum Associates, 1988.

[31] A. Tsiakiri , “Processing speed and attentional shift/mental flexibility in patients with stroke: A comprehensive review on the trail making test in stroke studies,” Neurol. Int., vol. 16, no. 1, pp. 210–225, Jan. 2024, doi: 10.3390/neurolint16010014. PMC10893544 38392955

[32] J. Kries, P. De Clercq, R. Lemmens, T. Francart, and M. Vandermosten, “Acoustic and phonemic processing are impaired in individuals with aphasia,” Sci. Rep., vol. 13, no. 1, Jul. 2023, Art. no. 11208. 10.1038/s41598-023-37624-w PMC10336064 37433805

[33] M. R. Love, “Auditory comprehension and working memory limits in aphasia,” Aphasiology, vol. 27, no. 7, pp. 843–869, 2013.

[34] R. Ratcliff, P. L. Smith, S. D. Brown, and G. McKoon, “Diffusion decision model: Current issues and history,” Trends Cognit. Sci., vol. 20, no. 4, pp. 260–281, Apr. 2016, doi: 10.1016/j.tics.2016.01.007. PMC4928591 26952739

[35] P. Schober, C. Boer, and L. A. Schwarte, “Correlation coefficients: Appropriate use and interpretation,” Anesthesia Analgesia, vol. 126, no. 5, pp. 1763–1768, 2018. 10.1213/ANE.0000000000002864 29481436

[36] F. Faul, E. Erdfelder, A.-G. Lang, and A. Buchner, “G*Power 3: A flexible statistical power analysis program for the social, behavioral, and biomedical sciences,” Behav. Res. Methods, vol. 39, no. 2, pp. 175–191, May 2007, doi: 10.3758/bf03193146. 17695343

